# Goblet Cell Adenocarcinoma With Heterotopic Ossification: A Rare Case Report and Review of Literature

**DOI:** 10.7759/cureus.8312

**Published:** 2020-05-27

**Authors:** Mohammed Alghamdi, Tiffani Mathew, Andrea Breaux, Harpreet Chopra

**Affiliations:** 1 Pathology, University of Louisville School of Medicine, Louisville, USA; 2 Pathology and Laboratory Medicine, University of Louisville, Louisville, USA

**Keywords:** heterotopic bone, ossification, goblet cell adenocarcinoma

## Abstract

Heterotopic bone formation is a rare phenomenon when associated with gastrointestinal neoplasms. Here we present a rare case of heterotopic ossification associated with goblet cell adenocarcinoma of the appendix, and a literature review of such cases associated with neoplasms within and out of the gastrointestinal tracts. We reviewed the clinical data and when available, immunohistochemical markers of osteoblastic differentiation. Our review shows similar findings to prior reports of apparent high association of heterotopic bone formation with neoplasms with mucinous features. Two, previously proposed main hypotheses of the mechanisms are reviewed. The unique feature about this case is that goblet cell adenocarcinoma was not reported previously in the setting of bone formation.

## Introduction

Heterotopic ossification is defined as the formation of lamellar ectopic bone in the soft tissue [1]. It is commonly seen in various anatomic locations including muscles and joints. It can be associated with clinical scenarios such as burns, prolonged bedridden states secondary to brain injuries, and certain orthopedic surgeries [1]. Males tend to have a higher incidence possibly due to differences in muscle mass, mechanism of injury, and hormonal status that affects osteogenesis [1-2]. Here, we report a case of heterotopic ossification associated with goblet cell adenocarcinoma of appendiceal origin. Studying the upregulation of chondrogenic and osteogenic genes in the injured soft tissue as well as the tumor cells/microenvironment has been previously performed by other investigators and may help us understand the pathogenesis of this phenomenon.

## Case presentation

Clinical history

**Figure 1:**
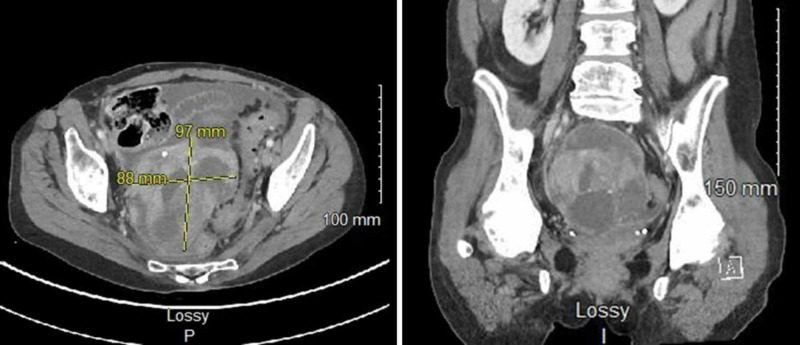
CT scan for abdomen and pelvis with contrast shows a large pelvic mass measuring 9.7 cm x 8.8 cm on the cross-sectional image (left), the coronal section showing lesional punctate hyperdensities compatible with ossification (right).

A 57-year-old female patient, presented with severe, intermittent abdominal pain, worsening in intensity. Abdominopelvic CT scan was performed and revealed a large pelvic mass in addition to small intestinal thickening (Figure 1). The patient was taken to the OR for total abdominal hysterectomy, bilateral salpingo-oophorectomy with tumor debulking. On intraoperative frozen section consultation, the ovary was involved by a malignant neoplasm. Histologic examination on paraffin-embedded sections showed an adenocarcinoma with signet ring cell features involving pelvic organs and omentum. Immunohistochemical studies were performed and showed immunoreactivity for CK20 and CDX2, consistent with a gastrointestinal primary. The gastrointestinal origin of this tumor was not suspected at the time of the surgery, hence the appendix was not resected. The oncology service started the patient on FOLFOX regimen (Folinic acid, Fluorouracil, Oxaliplatin). The patient completed a total of 12 cycles of FOLFOX with hyperthermic intraperitoneal chemotherapy (HIPEC) and underwent a right hemicolectomy after the eighth cycle of FOLFOX.

Surgical intervention and gross examination

**Figure 2:**
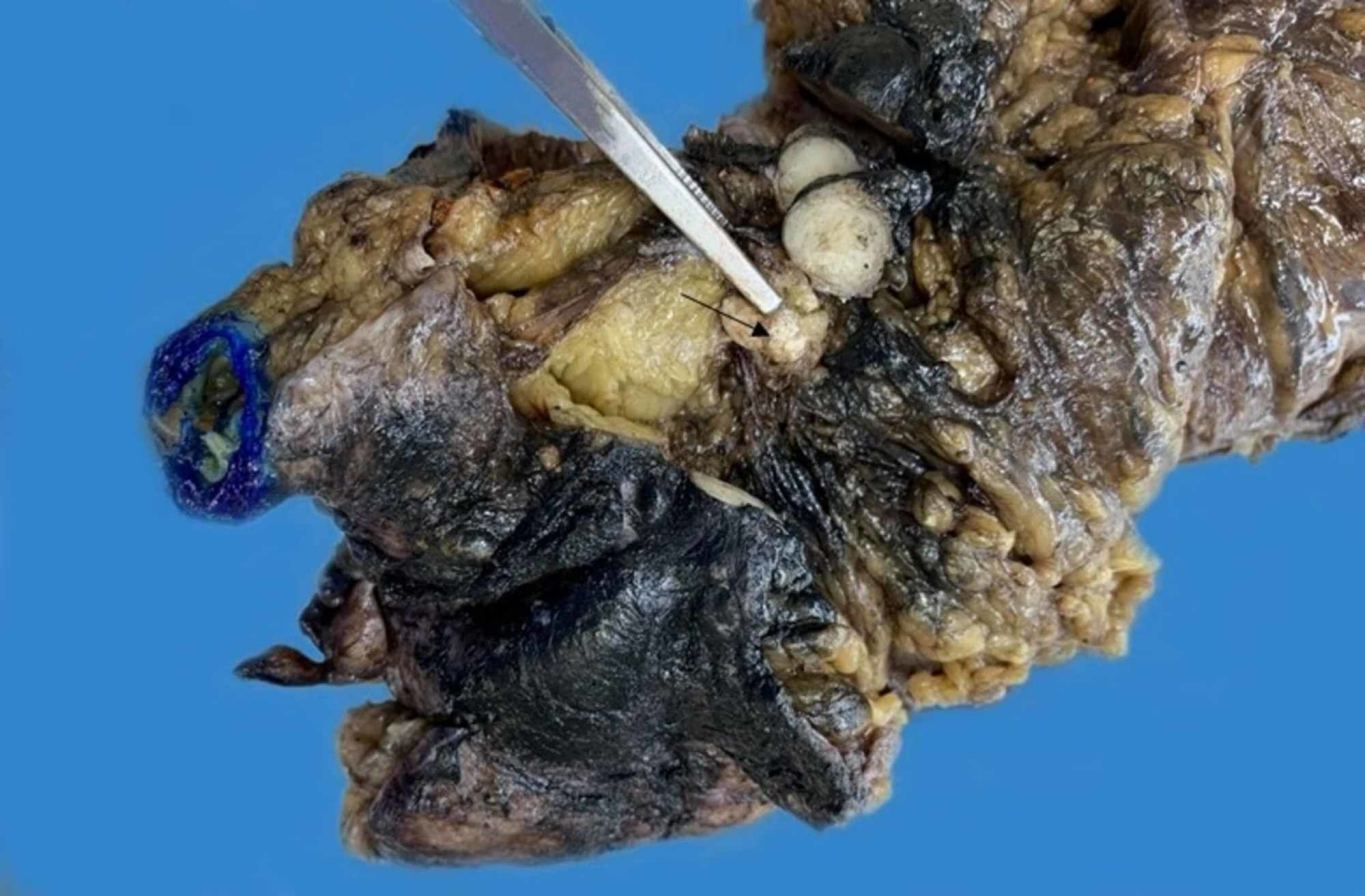
Gross photograph of the appendiceal tumor showing the obliterated lumen with calcification at the center (arrow).

The patient underwent an exploratory laparotomy, right hemicolectomy with en bloc omentectomy, cholecystectomy, and intraperitoneal tumor resection for her surgical management. The main specimen was received as a 30.5 cm segment of large bowel and terminal ileum with an attached appendix and a moderate amount of attached fibrofatty tissue. The appendix was adherent to the surrounding fibrofatty tissue and measured 5.0 cm in length x 1.2 cm in diameter. No tumor was grossly identified on the large bowel or ileal mucosa, or at the appendiceal orifice. Sectioning through the appendix revealed an obliterated lumen and a nodule that appeared to be contiguous with the appendiceal surface and wall. The nodule was markedly calcified and measured 2.5 cm in length x 0.9 cm in diameter and appeared to extend into the periappendiceal fat (Figure 2).

 

Histologic examination

**Figure 3:**
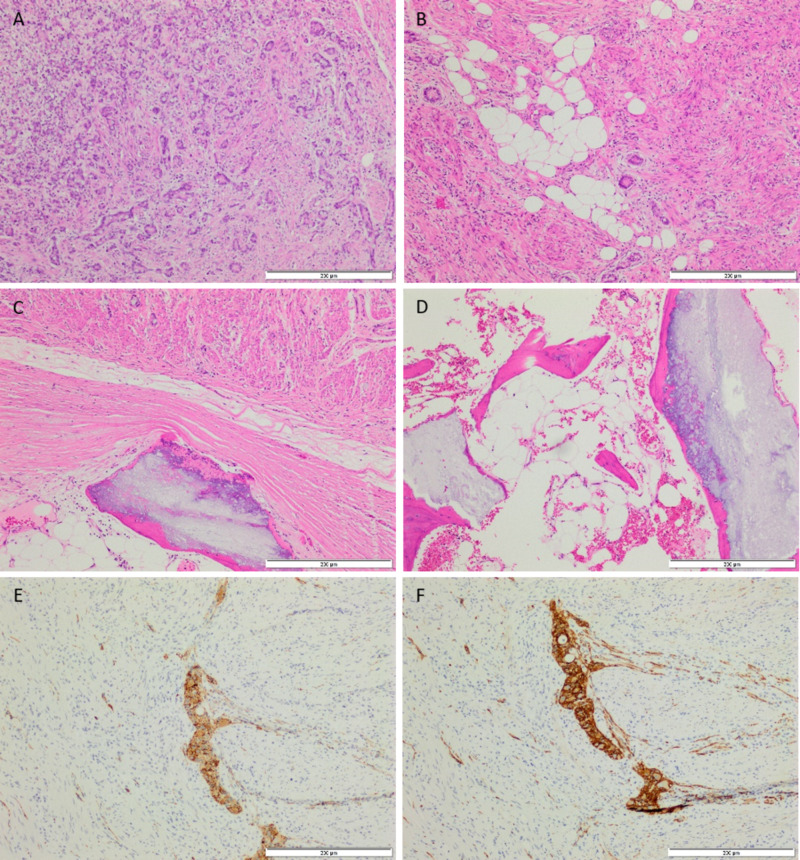
Photomicrographs of the appendiceal tumor. (A) Predominantly tubular proliferation of tumor cells with surrounding desmoplastic reaction. (B) Glandular elements and single cells invading the mesoappendix. (C, D) Formation of bone lined by osteoblasts. (E, F) Show tumor cells positive for synaptophysin and CD56, respectively. (Hematoxylin and Eosin stains and immunohistochemical studies, 20x)

Hematoxylin and eosin (H&E) stained slides showed a concentric transmural proliferation of tubules containing cells with minimal nuclear pleomorphism and a moderate amount of eosinophilic cytoplasm, along with goblet cells. The tumor cells were surrounded by a desmoplastic reaction and thickening of the appendiceal wall. The appendiceal lumen was obliterated with mature bone formation lined by osteoblasts. The tumor cells extended into the mesoappendix, as well as the adjacent small and large bowel, and there was an extensive perineural invasion. The goblet cells are positive for synaptophysin, chromogranin and CD56, and negative for CK7 (Figure 3).

## Discussion

Heterotopic bone formation is reported in association with tumors; some of which are summarized in Table 1. We present here the first reported case of heterotopic ossification in association with goblet cell adenocarcinoma of the appendix. The six case reports, in addition to our case of appendiceal tumors with mucin production and heterotopic ossification are summarized in Table 2. Various hypotheses have been put forth to explain heterotopic ossification in tumors. Huang et al. showed that the formation of bone in neoplastic settings results from pluripotent stromal cells undergoing osseous differentiation as highlighted by differential expression of morphogenic markers between the stroma and tumor cells [3]. Conversely, Noh et al. demonstrated the upregulation of chondrogenic and osteogenic markers in tumor cells favoring the idea of osteoblastic metaplasia of tumor cells [4].

**Table 1:** Summary of reported cases of neoplasia with heterotopic ossification and immunohistochemical studies performed. Gli2: glioma-associated oncogene family zinc finger 2, a-SMA: smooth muscle actin, BMP-2: bone morphogenetic protein 2, BMP-9: bone morphogenetic protein 9, bFGF: basic fibroblast growth factor, VEGF: vascular endothelial growth factor, TGF- β1: transforming growth factor, Runx2: Runt-related transcription factor 2.

Organ	Author	Tumor type	IHC markers tested
Colon	Huang et al. (2014) [3]	Adenocarcinoma	Osteonectin, Gli2, Nestin, a-SMA
Lung	Suzuki et al. (2019) [5]	Adenocarcinoma	BMP-2, Osteopontin
Rectum	Kypson et al. (2003) [6]	Adenocarcinoma	Osteocalcin, Osteopontin, β-catenin, BMP9
Thyroid	Takeda et al. (2013) [7]	Papillary thyroid carcinoma	bFGF, BMP-2, VEGF
Lung	Kim et al. (2009) [8]	Adenocarcinoma	TGF-β1, Osteopontin, Osteocalcin, Runx2
Lung	Tsubochi et al. (2013) [9]	Carcinoid tumor	BMP-2, Osteocalcin
Rectum	Smajda et al. (2015) [10]	Adenocarcinoma	Not performed

 

**Table 2:** Reported cases of neoplasia from the appendix with heterotopic ossification. BMP9: bone morphogenetic protein 9, ALK1: anaplastic lymphoma kinase 1

Case	Age (sex)	Past medical history	Diagnosis	Size (cm)	IHC
Juvara et al. (1948) [11]	70 (M)	Strangulation of a right inguinoscrotal hernia	Perforated mucocele	7.0	NA
Haque et al. (1996) [12]	46 (F)	Chronic ulcerative colitis, in remission	Mucinous cystadenocarcinoma	2.0	NA
Choi et al. (2016) [13]	44(F)	Papillary thyroid carcinoma	Mucinous cystadenoma	3.5	NA
Choi et al. (2016) [13]	56 (F)	Abdominal pain	Low-grade appendiceal mucinous neoplasm	7.0	NA
Choi et al. (2016) [13]	58 (F)	Incidental pelvic mass	Mucinous cystadenocarcinoma	8.0	NA
Noh et al. (2016) [4]	72 (F)	Dyspepsia, weight loss, hyperlipidemia	Perforated low-grade appendiceal mucinous neoplasm	8.0	BMP9, ALK1, Osteocalcin, Osteopontin
Current case	57 (M)	Abdominal pain	Adenocarcinoma ex goblet cell carcinoid	5.0	NA

## Conclusions

As more cases are reported, a probable mechanism for heterotopic ossification associated with neoplasia is gaining better understanding. Further studies to assess the clinical significance of bone formation in these tumors, and possible correlation with tumor behavior are warranted. One of the limitations of this case report is the lack of morphoproteomic analysis via immunohistochemistry due to financial reasons.
